# An Approach to the Stepwise Management of Severe Mitral Regurgitation with Optimal Cardiac Pacemaker Function

**DOI:** 10.1016/s0972-6292(16)30732-x

**Published:** 2014-03-12

**Authors:** Christopher V DeSimone, Vuyisile T Nkomo, Daniel C DeSimone, Lawrence R Keenan, Maurice Enriquez-Sarano, Samuel J Asirvatham

**Affiliations:** 1 Division of Cardiovascular Diseases and Internal Medicine, Mayo Clinic, Rochester, MN; 2 Division of Infectious Diseases and Internal Medicine, Mayo Clinic, Rochester, MN; 3 Division of Pediatrics and Adolescent Medicine, Mayo Clinic, Rochester, MN

**Keywords:** mitral regurgitation, cardiac pacemaker

## Abstract

Right ventricular apical pacing may cause or worsen mitral regurgitation (MR). Potential mechanisms for this adverse sequelae include intraventricular dyssynchrony, altered papillary muscle function, pacing-induced cardiomyopathy with left ventricular dilation, and annular dilation. In contrast, biventricular (BiV) pacing may improve MR presumably by opposing the negative effects. Whether or not left ventricular lead location is important in treating mitral regurgitation in patients with pacemakers is unknown.

We report a case of severe MR and left ventricular (LV) systolic failure in a patient with right ventricular pacing. Multiple potential etiologies for the worsening valve function were noted, and a stepwise iterative optimizing scheme that included basal lateral LV pacing improved mitral valve function and ameliorated heart failure symptoms.

## Introduction

Mitral regurgitation (MR) can worsen as a result of acute or chronic right ventricular apical pacing [1,2]. The mechanism is thought to be a consequence of intraventricular dyssynchrony and altered papillary muscle mechanics [3]. In contrast, bi-ventricular pacing may improve mitral regurgitation by decreasing left ventricular chamber size during systole [4] and improvement in mitral annular contraction [5]. Both of these mechanisms would have the effect of limiting dilation of the mitral annular apparatus and thus improving valve function.

We report a case of worsening mitral regurgitation and systolic heart failure caused by right ventricular apical pacing. A cardiac resynchronization device was ultimately placed for biventricular pacing and improved severe MR, systolic function, and heart failure symptoms.

This case illustrates that even after optimization of pacemaker function for improved contractility and valve function; a real incremental benefit can be seen with a very basal positioning of the LV lead.

## Case History

**Figure 1A and 1B:**
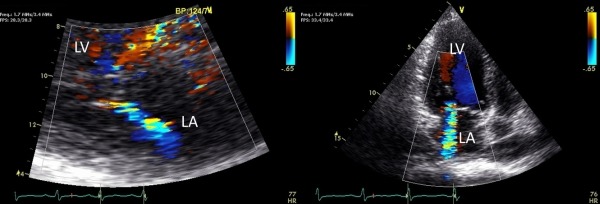


An 85-year-old female with a history of hypertension developed symptomatic paroxysmal atrial fibrillation manifesting as palpitations associated with dyspnea, weakness, and presyncope. Holter monitoring confirmed paroxysmal atrial fibrillation (PAF) with rapid ventricular response and echocardiography showed a mild reduction in left ventricular systolic function with an ejection fraction of 49%, diastolic dysfunction, and mild to moderate mitral regurgitation (MR) (Figure 1A and 1B). Rate control was unsuccessful and resulted in multiple bouts of heart failure and recurrent visits to the emergency room. The patient was scheduled for atrioventricular node ablation and dual chamber pacemaker implantation the following week.

Ablation and pacemaker implantation was successful. The pacemaker was initially programmed to DDDR but due to a large amount of pacing energy required was programmed to VVIR. At 3 month device follow up, her atrial lead thresholds improved and the pacemaker mode was changed to DDDR.

**Figure 2A and 2B:**
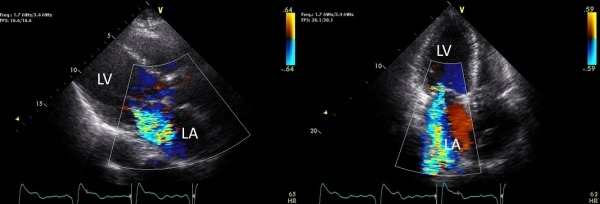


Approximately two weeks after pacemaker settings were changed, she presented in decompensated heart failure. A transthoracic echocardiography showed worsening left ventricular systolic function with ejection fraction decreasing to 30%, moderate to severe decreased right ventricular function and RV enlargement, and development of severe MR (Figure 2A and 2B). She was medically managed to optimize her heart failure medications, but continued to decompensate over the next 2-3 months. Her device was interrogated and pacemaker was not mode switching with atrial arrhythmia. This led to atrial tracking at a high rate and thus rapid ventricular pacing during AF, resulting in worsening heart failure symptoms. Mode tracking function of pacemaker was found to be "off". Her device settings were modified and mode switch was turned on. This allowed her to switch from ventricular tracking of AF to VVIR.

**Figure 3A and 3B:**
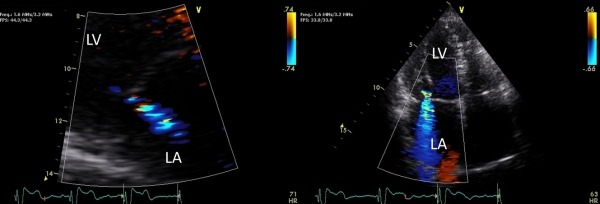


Pacemaker reprogramming resulted in only slight improvement in overall LVEF to 35% at one month follow up, but left ventricular contraction was dyssynchronous and severe MR persisted. The patient continued to have refractory brittle congestive heart failure. A potential cause of MR such as coronary ischemia was evaluated with angiography but only showed mild coronary artery disease. She was on optimal medical management with beta-blocker, two diuretics, and an ACE inhibitor. However, because of left ventricular dyssynchrony and severe MR on repeat echo at 3 months, the decision was made to upgrade the pacemaker to a biventricular pacing system. The left ventricular lead was placed in the coronary sinus at the mid-basal position. Two months later, echocardiography revealed improved left ventricular synchrony, contractility, and left ventricular ejection fraction increased to 40%, RV systolic function improved back to baseline, and there was significant improvement of MR back to baseline mild to moderate (Figure 3A and 3B). The patient's symptoms improved and she has had no recurrent bouts of heart failure to date.

## Discussion

Pacemaker-induced worsening MR and the subsequent improvement in valvular function by altering pacing has been highlighted in the literature. The improvements in valvular regurgitation have come about through altering the site of right ventricular lead pacing to the interventricular septum [6], or placement in the right ventricular outflow tract [2]. Changing mode of pacing from VVI to atrial pacing with intrinsic ventricular rhythm has also been reported to improve MR [7]. Upgrading to resynchronization therapy has proven beneficial in patients with acute worsening of MR from pacemaker induced dyssynchrony with [8,9] and without ischemic heart disease [10].

Several key points are illustrated by our case. First, this case highlights the importance of pacing mode post nodal ablation in patients with baseline valvular disease and decreased cardiac function [11]. Second, biventricular pacing is a viable option for patients with systolic dysfunction who develop worsening mitral regurgitation due to right ventricular apical dyssynchronous pacing. Third, our case underscores how synchronicity of pacing can improve cardiac contractility and mitigate heart failure symptoms in patients with biventricular heart disease. Fourth, even after a stepwise approach was taken to improve heart function through pacemaker optimization, a dramatic improvement in mitral valve function can occur with a lead positioning strategy. One hypothesis that merits further research is that improvement in MR occurs because basal lead positioning causes earlier contraction of the mitral annulus and thus improves MV mechanical synchrony.

## References

[R1] Alizadeh A (2011). Induction and aggravation of atrioventricular valve regurgitation in the course of chronic right ventricular apical pacing. Europace.

[R2] Miranda R (2010). Acute severe mitral regurgitation as an early complication of pacemaker implantation. Europace.

[R3] Kanzaki H (2004). A mechanism for immediate reduction in mitral regurgitation after cardiac resynchronization therapyInsights from mechanical activation strain mapping. Journal of the American College of Cardiology.

[R4] Vinereanu D (2007). Mechanisms of Reduction of Mitral Regurgitation by Cardiac Resynchronization Therapy. Journal of the American Society of Echocardiography.

[R5] Ypenburg C (2008). Mechanism of improvement in mitral regurgitation after cardiac resynchronization therapy. European Heart Journal.

[R6] Stepowski D (2011). Significant mitral regurgitation regression by slight modification of the right ventricular pacing site. Europace.

[R7] Wong DTL (2010). Severe mitral regurgitation due to right ventricular apical pacing. BMJ Case Reports.

[R8] Haider AS (2008). Acute Pulmonary Edema Due to Pacemaker-Induced Mitral Regurgitation. Journal of Invasive Cardiology.

[R9] Disney PJS (2003). Biventricular Pacing for Severe Mitral Reguritation Following Atrioventrgicular Nodal Ablation. Pacing and Clinical Electrophysiology.

[R10] de Guillebon MP (2007). Regression of mitral regurgitation after cardiac resynchronization therapy in an adult with preserved left ventricular function and right ventricular pacing. Europace.

[R11] Curtis AB (2013). Biventricular Pacing for Atrioventricular Block and Systolic Dysfunction. New England Journal of Medicine.

